# Corticosteroid injection or dry needling for musculoskeletal pain and disability? A systematic review and GRADE evidence synthesis

**DOI:** 10.1186/s12998-021-00408-y

**Published:** 2021-12-02

**Authors:** Luis Fernando Sousa Filho, Marta Maria Barbosa Santos, Gabriel Henrique Freire dos Santos, Walderi Monteiro da Silva Júnior

**Affiliations:** 1 Graduate Program in Physical Education, Federal University of Sergipe, Av Marechal Rondon, s/n, Rosa Elze, São Cristovão, Sergipe, 49100-000 Brazil; 2 Department of Physical Therapy, Federal University of Sergipe, Av Marechal Rondon, s/n, Rosa Elze, São Cristovão, Sergipe, 49100-000 Brazil

**Keywords:** Pain, Disability, Steroids, Acupuncture, Manual therapy

## Abstract

**Background:**

Corticosteroid injection and dry needling have been used in the treatment of musculoskeletal conditions, but it is unclear which intervention is the most effective. The purpose of this study was to compare the effects of corticosteroid injection and dry needling for musculoskeletal conditions at short-, medium-, and long-term follow-up.

**Methods:**

Electronic databases were searched up to 31 October 2021. Two researchers independently screened titles, abstracts and full-text articles. Randomized clinical trials (RCTs) that investigated the effectiveness of dry needling compared to corticosteroid injection in patients over 18 years with a musculoskeletal condition were included in the review. The studies had to report pain and/or disability as outcome. Risk of bias was assessed by using the revised Cochrane Collaboration tool (RoB 2.0). Quality of evidence was evaluated by using the GRADE approach.

**Results:**

Six studies were included (n = 384 participants). Four musculoskeletal conditions were investigated. There is very low-quality evidence that CSI is superior to DN for reducing heel pain (plantar fasciitis) and lateral elbow pain at short- and medium-term follow-up, but not for myofascial pain and greater trochanteric pain. There is very low-quality evidence that DN is more effective than CSI at long-term follow-up for reducing pain in people with plantar fasciitis and lateral epicondylitis. Very low-certainty evidence shows that there is no difference between DN and CSI for disability at short-term follow-up. One study showed that CSI is superior to DN at medium-term follow-up and another observed that DN is superior to CSI for reducing disability at long-term.

**Conclusions:**

There are no differences between DN and CSI in pain or disability for myofascial pain and greater trochanteric pain syndrome. Very-low certainty evidence suggests that CSI is superior to DN at shorter follow-up periods, whereas DN seems to be more effective than CSI at longer follow-up durations for improving pain in plantar fasciitis and lateral epicondylitis. Large RCTs with higher methodological quality are needed in order to draw more incisive conclusions.

***PROSPERO registration number*:**

CRD42020148650.

**Supplementary Information:**

The online version contains supplementary material available at 10.1186/s12998-021-00408-y.

## Background

Corticosteroid injections (CSI) have been widely used in the management of musculoskeletal conditions in order to reduce inflammation, pain and disability [1]. Corticosteroids are exogenous drugs that mimic the endogenous steroid hormones and are involved in physiological process such as the regulation of metabolism, skeletal growth and immune function [2]. The anti-inflammatory effects of corticosteroids may be due to a down-regulation of pro-inflammatory cytokines and genes [2]. As inflammation is involved in pain-related mechanisms, corticosteroid injections are useful to reduce pain and, consequently, disability.

Although some studies have found positive effects of corticosteroid injections for common musculoskeletal conditions, other trials have found no clinically meaningful improvements in comparison to placebo injections [3–5]. In addition, corticosteroid injections cause adverse effects [6, 7]. It has been showed that local inflammation may increase up to three days following corticosteroid application, as well as adrenal suppression and cartilage damage [6]. A randomized clinical trial (RCT) observed that an intra-articular corticosteroid injection caused loss of cartilage without reducing pain at two years follow-up [7]. These findings suggest that corticosteroid injections may be used with caution.

An alternative option to the use of corticosteroid injections is dry needling (DN). It consists of a needling stimulation without drugs that can be used in various body areas aiming to reduce pain and disability. Although its mechanisms are not fully understood, it has been suggested that a local twitch response provoked by dry needling may send neural inputs to the brain that would help to break the vicious cycle of pain-spasm-pain [8, 9]. Dry needling stimulation is suggested to reduce the nociceptive output in different tissues by improving blood flow, increasing fibroblastic activity and modulating central mechanisms [8].

A previous meta-analysis has shown that dry needling is superior to control/sham dry needling for pain and functional outcomes in individuals with musculoskeletal conditions [11]. However, the observed differences were not considered to be clinically meaningful for pain outcomes. Whereas the treatment mean difference for dry needling on pain scores was 1.27 points, an expected clinically meaningful change would be superior to a 2-point change on the Visual Analogue Scale (VAS) [11]. Compared to other treatments such as soft tissue manual therapy interventions, dry needling exhibited better improvements on pain intensity and pain pressure threshold at a 12 weeks follow-up [11]. In addition to its clinical effects, there are reports of side effects following dry needling therapy. Alternatively, a survey with physiotherapists has only reported mild adverse events related to dry needling intervention such as bruising, bleeding and pain during/after treatment [12]. Reports of aggravating symptoms, fatigue, nausea, and numbness were uncommon. Another survey has found similar results [13]. Although minor adverse events were reported in over a third of DN treatment sessions, major adverse events were rare (< 0.1%) [13].

The effect of corticosteroid injections and dry needling for musculoskeletal conditions varies at different timepoints [3, 5, 8, 11]. Most studies have found positive findings for both interventions at short-term follow-up. It has been suggested that corticosteroid injections are more effective at short-term because their effects are associated with the short half-live of the corticosteroids that are injected [14]. In this way, dry needling would be more effective than corticosteroid injections at longer follow-up assessments. Although it is already known that corticosteroid injections and dry needling interventions change pain and disability outcomes in people with musculoskeletal conditions [3, 5, 8, 11], no previous systematic reviews were found in order to summarize and to compare the effects of these interventions at different follow-up periods.

It is relevant to investigate the effects of dry needling and corticosteroid injections for musculoskeletal conditions because these techniques have been used routinely in the primary care regardless of the recommendations from evidence-based clinical practice guidelines. More than 50% of physiotherapists responding to a survey in the United States reported to use dry needling in their clinical practice [15]. In addition, a previous study showed that steroid injection is the second most used therapy for managing shoulder pain by Australian general practitioners [16]. Despite the frequent use of these therapies, clinical practice guidelines for musculoskeletal conditions have not usually mentioned neither dry needling nor corticosteroid injections as first line treatment [17–21]. Some guidelines have recommended dry needling and corticosteroid injection as adjunct treatment for some musculoskeletal conditions such as plantar fasciitis and Achilles pain, but it is suggested to be used with caution [18, 19, 21].

Given that (1) both corticosteroid injection and dry needling have been extensively used to manage musculoskeletal conditions, (2) it is unclear if one intervention is superior over the other for pain and disability outcomes, and (3) the effect of these interventions at different time points is under investigated, the aim of this systematic review was to compare the effect of these techniques on pain and disability in individuals with musculoskeletal conditions at short-, medium- and long-term follow-up.

## Methods

### Eligibility criteria

This review was registered with PROSPERO (CRD42020148650) and has been reported according to the Preferred Reporting Items for Systematic Reviews and Meta-Analyses (PRISMA) guidelines [22].

Included studies were randomized clinical trials (RCTs), fastidious or pragmatic, that investigated the effects of dry needling compared to corticosteroid injection. Tenotomy was not considered. Participants had to have musculoskeletal pain and to be over 18 years. Musculoskeletal pain was defined as pain located in the muscles, bone, joints, or tendons such as low back and neck pain [23]. This review considered both localized and widespread musculoskeletal pain regardless the duration of pain. The outcome had to be pain and/or disability. Studies using oral corticosteroids (not injectable) were excluded. Pilot studies and conference abstracts were also excluded. Trials using corticosteroid mixture injections and trials allowing co-interventions were included. No language or follow-up restriction was applied.

### Information sources and search strategy

Search was conducted by two independent researchers in the following databases (from earliest record to 31 October 2021): MEDLINE (Pubmed), SCOPUS (Elsevier), CINAHL (EBSCO), SPORTDiscus (EBSCO) and Web of Science (Clarivate analytics). Search strategy included keywords related to ‘dry needling’ and ‘corticosteroids’ using Booleans operators. Searches were not peer reviewed. The full search strategy is described in the Appendix (available as Additional file 1). Citation tracking (hand search of the reference list of eligible studies and published relevant reviews) was performed. Grey literature (http://www.opengrey.eu and http://oaister.worldcat.org/) and study registers (https://clinicaltrials.gov/) were searched.

### Selection process

The selection of eligible studies was performed by two investigators independently that screened titles, abstracts and full-text articles to achieve a consensus on which studies to select. Only one investigator performed data extraction from the included studies using a self-developed screening form. Then, the second investigator checked the extracted data. A third investigator resolved any disagreement. Extracted information included study characteristics (author, year, condition, funding source, conflict of interest), methods (study design, data collection period, country), participant’s characteristics (sample size, sex, age), intervention details (type of intervention, dose and frequency), outcomes measures and time points (follow up), results, adverse events and responder analysis, when reported. When necessary, missing data were requested from study authors.

### Risk of bias assessment

To assess the risk of bias, the revised Cochrane Collaboration tool for assessing risk of bias (Risk of Bias 2.0) in randomized clinical trials was used [24]. This tool evaluates five domains: bias arising from the randomization process, bias due to deviations from intended intervention, bias due to missing outcome data, bias in measurement of the outcome, and bias in selection of the reported result. Two independent investigators made the judgment of each item as at either ‘low risk’, ‘high risk’ or ‘some concerns’. Any disagreement was resolved by discussion between the investigators. If consensus was not achieved by discussion, a third investigator opinion was sought.

### Certainty assessment

The quality of evidence for each outcome was assessed by using the Grading of Recommendations Assessment, Development and Evaluation (GRADE) approach [25]. Five factors were considered in the overall quality evaluation: risk of bias (≥ 25% of the trials were judged as at high risk of bias), inconsistency (I^2^ ≥ 50% or if pooling was not possible), indirectness (≥ 50% of the trials presented differences in study population, interventions or outcome measures), imprecision (< 400 participants per outcome) and publication bias (assessed by funnel plot asymmetries whether there are ten studies or more for each outcome). For each factor judged to be present, the quality of evidence was downgraded one level. Overall quality of evidence levels were classified into high, moderate, low or very low quality.

### Data synthesis

Data analysis was based on the follow-up period (short-term: ≤ 6 weeks; mid-term: 7–23 weeks; and long-term: ≥ 24 weeks). Results were expressed as mean ± SD for each measure. When outcomes scales differed from the direction of the effect, we have multiplied the mean values of the differing scales by − 1. Meta-analysis was not performed due to the high level of heterogeneity across studies.

## Results

**Fig. 1:**
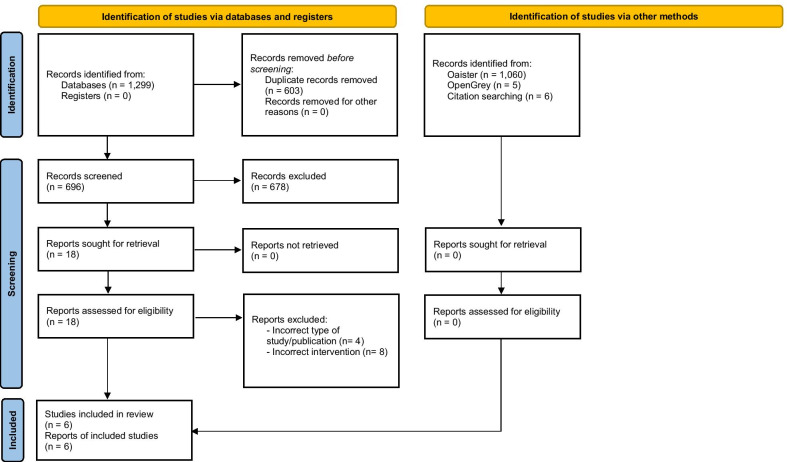
PRISMA flowchart

A total of 1,299 studies were identified from databases in the initial search. After duplicates were removed, 696 remained. Of these, 18 were retrieved for full-text screening. Six studies were included in the final analysis [26–31]. Although 1071 studies were identified from other sources, none was retrieved for full-text screening (Fig. 1).

### Study characteristics

The included studies involved 384 participants with 190 enrolled in DN group and 194 enrolled in CSI group. The characteristics of the studies are shown in Table 1. The majority of the participants were female and the estimated average age was 49 years old. Two studies did not report data for sex of the participants [29, 30]. Musculoskeletal conditions assessed were plantar fasciitis (2 trials) [27, 28], greater trochanteric pain syndrome (1 trial) [26], lateral epicondylitis (2 trials) [30, 31] and myofascial pain with associated headache (1 trial) [29]. These conditions were chronic (symptoms > 3 months) in four trials. One trial did not limit the duration of symptoms [29]. No conflict of interest was reported.

**Table 1:** Characteristics of the included studies

Author, Year Condition Funding source CoI	Methods	Participants	Interventions	Outcomes measures and time points	Results (outcomes, responder analysis, adverse events)
Güngör and Güngör [31] Lateral epicondylitis Not funded None	Randomized clinical trial conducted in Turkey from 2018 to 2020	Sample size (n)* DN: 24 CSI: 24 Sex (Females) DN: 13F CSI: 15F Age (mean ± SD) DN: 46.0 ± 7.4 years CSI: 40.9 ± 7.7 years *This study had another intervention group, which received platelet-rich plasma (PRP) treatment (n = 24)	DN: A fine needle (23 gauge) was withdrawn and advanced throughout the long axis of the tendon about 40–50 times during 2 min to pepper the tendon. Dry needling was performed once a week for 3 weeks CSI: A single corticosteroid injection of 40 mg methylprednisolone acetate was administered with the patient seated on a chair and the forearm placed in the neutral position and the elbow in 90º of flexion	Pain intensity measured by using the Visual Analogue Scale (VAS) and function assessed through Disabilities of the Shoulder, Arm, and Hand (DASH) score before treatment, 3 weeks and 3 months follow-up	Pain (VAS) 3 wk: DN 2.3 ± 0.6 versus CSI 2.3 ± 0.6 p = 0.98 3mo: DN 1.1 ± 0.5 versus CSI 0.7 ± 0.6 p = 0.01 Function (DASH) 3wk: DN 31.6 ± 6.8 versus CSI 32.0 ± 5.0 p = 0.84 3mo: DN 30.0 ± 6.7 versus CSI 26.6 ± 3.2 p = 0.01 Responder analysis: Not reported Adverse events: Not reported
Rastegar et al. [27] Plantar Fasciitis Medical Research Council at Isfahan University of Medical Sciences None	Randomized clinical trial conducted in Iran from April 2013 to April 2015	Sample size (n) DN: 32 CSI: 34 Sex (Females) DN: 18F CSI: 20F Age (mean ± SD) DN: 39.8 ± 7.9 years CSI: 42.0 ± 10.3 years	DN: The medial aspect of heel pad through the point of maximum tenderness was dry needled for 30 s using a 0.3 mm diameter needle. The needle was withdrawn and advanced during the time CSI: 1 ml of methylprednisolone acetate (40 mg/ml) was injected to the medial aspect of heel pad through the point of maximum tenderness using a 0.3 mm diameter needle with 3 or 2 ml syringe	Pain intensity measured by VAS before treatment, 3 weeks, 6 weeks, 3 months, 6 months and 1 year after treatment	Pain (VAS) Short-term 3wk: DN 3.47 ± 1.32 versus CSI 0.32 ± 0.71 p < 0.001 6wk: DN 2.66 ± 1.33 versus CSI 0.21 ± 0.67 p < 0.001 Mid-term 12wk: DN 1.59 ± 1.24 versus CSI 0.56 ± 1.33 p = 0.44 Long term 6mo: DN 1.28 ± 1.46 versus CSI 1.79 ± 1.55 p = 0.65 1 yr: DN 0.69 ± 0.93 versus CSI 2.09 ± 1.58 p = 0.004 Responder analysis: Not reported Adverse events: Not reported
Brennan et al. [26] Greater trochanteric pain syndrome Baylor Scott & White Health None	Prospective, randomized clinical trial conducted in USA between 2013 and 2015	Sample size (n) DN: 21 CSI: 22 Sex (Females) DN: 19F CSI: 18F Age (mean ± SD) DN: 61.3 ± 16.5 years CSI: 70.1 ± 11.4 years	DN: Muscles assessed as contributing for patient pain was needled by a 0.3–0.5 mm diameter needle. The needle was withdrawn and advanced repeatedly while it produced an appropriate response and was tolerated by the participant. Then, the needle was left in situ for 5–7 min CSI: A 10 ml injection mixture containing 2 mL methylprednisolone acetate (40 mg/mL), 4 mL 1% lidocaine and 4 mL 0.25% marcaine was injected in the point of maximal tenderness on the greater trochanter with an area coverage of 3-4 cm by 3–4 cm. 2 mL were injected perpendicular to the skin to the level of bony contact; then, 2 mL were injected in each of 4 quadrants around the injection side. Needle length was 1.5 inch and diameter was 21 or 22 gauge, depending on soft tissue thickness	Pain intensity measured by NPRS and function measured by PSFS before treatment, 1 week, 3 weeks and 6 weeks after treatment	Pain (NPRS) Short term 1wk: DN 3.6 ± 2.1 versus CSI 2.6 ± 2.7 3wk: DN 4.0 ± 2.2 versus CSI 2.7 ± 2.9 6wk: DN 2.8 ± 2.4 versus CSI 3.9 ± 3.7 Disability (PSFS)Short term 1wk: DN 5.2 ± 2.2 versus CSI 6.5 ± 2.8 3wk: DN 5.7 ± 2.0 versus CSI 6.5 ± 2.8 6wk: DN 7.3 ± 2.3 versus CSI 6.1 ± 3.0 Responder analysis: Not reported Adverse events DN: 0% (n = 0) CSI: 0% (n = 0)
Uygur et al. [30] Lateral epicondylitis Not reported None	Randomized clinical trial conducted in Turkey,	Sample size (n) DN: 49 CSI: 52 Sex (Females) – Age (mean ± SD) DN: 47.5 ± 7.3 years CSI: 48.1 ± 10.3 years	DN: Fifteen 0.25 × 25 mm needles were inserted at the lateral epicondyle region. The needles were rotated 3–4 times, held in place for 10 min, and withdrawn. Dry needling was performed twice a week for 5 sessions CSI: A single dose of corticosteroid injection containing 2 ml methylprednisolone acetate (40 mg/ml) was inserted into the lateral epicondyle region. The periosteum was pricked 90 20–30 times without withdrawing the needle and no local anesthetic was used	Patient improvement was assessed by using the Patient-rated Tennis Elbow Evaluation (PRTEE) before interventions, at 20th day and at 6 months follow-up	Patient improvement 20th day: DN 15.6 ± 7.7 versus CSI 36.0 ± 14.7 p < 0.01 6 months: DN 9.7 ± 7.6 versus CSI 19.3 ± 19.4 p < 0.01 Responder analysis: not reported Adverse events DN: 2% (n = 1) of participants did not tolerate pain due to intervention and was excluded from the study CSI: 7.6% (n = 4) of participants presented skin atrophy and whitening at 6-months follow-up
Uygur et al. [28] Plantar fasciitis Not funded None	Randomized clinical trial conducted in Turkey	Sample size (n) DN: 49 CSI: 47 Sex (Females) N(%): 63F (66%)* Age (mean ± SD) DN: 49.6 ± 11.7 years CSI: 49.9 ± 12.3 years	DN: Fifteen-0.25 × 0.4 mm needles were inserted into the plantar fascia origin at the calcaneous. The needles were left in situ for 10 min and were rotated 3 to 4 times. Dry needling was performed twice a week for 5 sessions CSI: A single dose of corticosteroid injection containing methylprednisolone acetate (2 mL Depo Medrol, 40 mg/ml) and bupivacaine (1 mL marcaine, 0.5%) was injected between the plantar fascia and the periosteum. Needle diameter was 25 gauge	Pain intensity and disability score measured by the Foot Function Index (FFI) Before treatment, 3 weeks and 6 months after treatment	Pain (FFI Pain score) Short term 3wk: DN 27.7 ± 9.82 versus CSI 33.6 ± 10.6 Long term 6mo: DN 29.7 ± 10.3 versus CSI 50.5 ± 12.3 Disability (FFI Disability Score) Short term 3wk: DN − 28.3 ± 8.9 versus CSI − 28.4 ± 11.6* Long term 6mo: DN − 28.8 ± 8.8 versus CSI − 43.1 ± 11.1* Responder analysis: Not reported Adverse events DN: 38% (n = 18) of participants presented pain at the needling site and 12% (n = 6) reported subcutaneous bleeding CSI: 0% (n = 0) *multiplied by − 1 to adjust the direction of the effect
Venancio et al. [29] Myofascial pain and headache Not reported Not reported	Randomized clinical trial conducted in Brazil	Sample size (n) DN: 15 CSI: 15 Sex (Females) – Age (mean ± SD) –	DN: not sufficiently described CSI: Corticosteroid injection mixture containing 0.2 mL dexamethasone (4 mg/mL) and lidocaine (0.25%)	Pain measured by the modified Symptom Severity Index (SSI) Before treatment, 1 week, 4 weeks and 12 weeks after treatment	Pain (Modified SSI) Short term 1wk: DN 0.34 ± 0.08 versus CSI 0.51 ± 0.20 4wk: DN 0.42 ± 0.08 versus CSI 0.43 ± 0.11 Mid term 12wk: DN 0.36 ± 0.17 versus CSI DN 0.33 ± 0.1 Responder analysis: Not reported Adverse events: Not reported

*CoI* conflict of interest, *CSI* corticosteroid injections, *DN* dry needling, *NPRS* numerical pain rating scale, *PSFS* patient specific functional scale, *USA* United States of America

*It refers to the proportion of females in the entire sample. The study has not reported the proportion of females for each intervention group

All studies had pain as an outcome. Three studies investigated disability. One study has used the Patient Reported Tennis Elbow Evaluation (PRTEE) tool, which evaluates both pain and disability [30]. Pain was assessed by using different scales: Foot Function Index (FFI) [28], Visual Analogue Scale (VAS) [27], Numeric Pain Rating Scale (NPRS) [26], and modified Symptom Severity Index (SSI) [29]. Disability was assessed though FFI [28], Disabilities of the Shoulder, Arm and Hand (DASH) [31] and Patient-Specific Functional Scale (PSFS) [26]. Short-term effects were assessed by all studies and medium- and long-term effects were evaluated by five studies [27–31]. Dosage and type of corticosteroid injection varied across the studies. Three studies used a corticosteroid mixture containing anesthetics [26, 28, 29]. For dry needling intervention, the needle diameter and the technique parameters (time left in situ, puncture pattern) were different across the studies.

All studies used one single dose application of corticosteroid injections. For dry needling intervention, Rastegar et al. [27] and Venancio et al. [29] used one single session of treatment, Uygur et al. [28, 30] used 5 sessions (twice a week), Güngör et al. [31] used 3 sessions (once a week) and Brennan et al. [26] used between 3 and 7 sessions (at discretion of the therapist).

### Risk of bias and outcomes

**Fig. 2:**
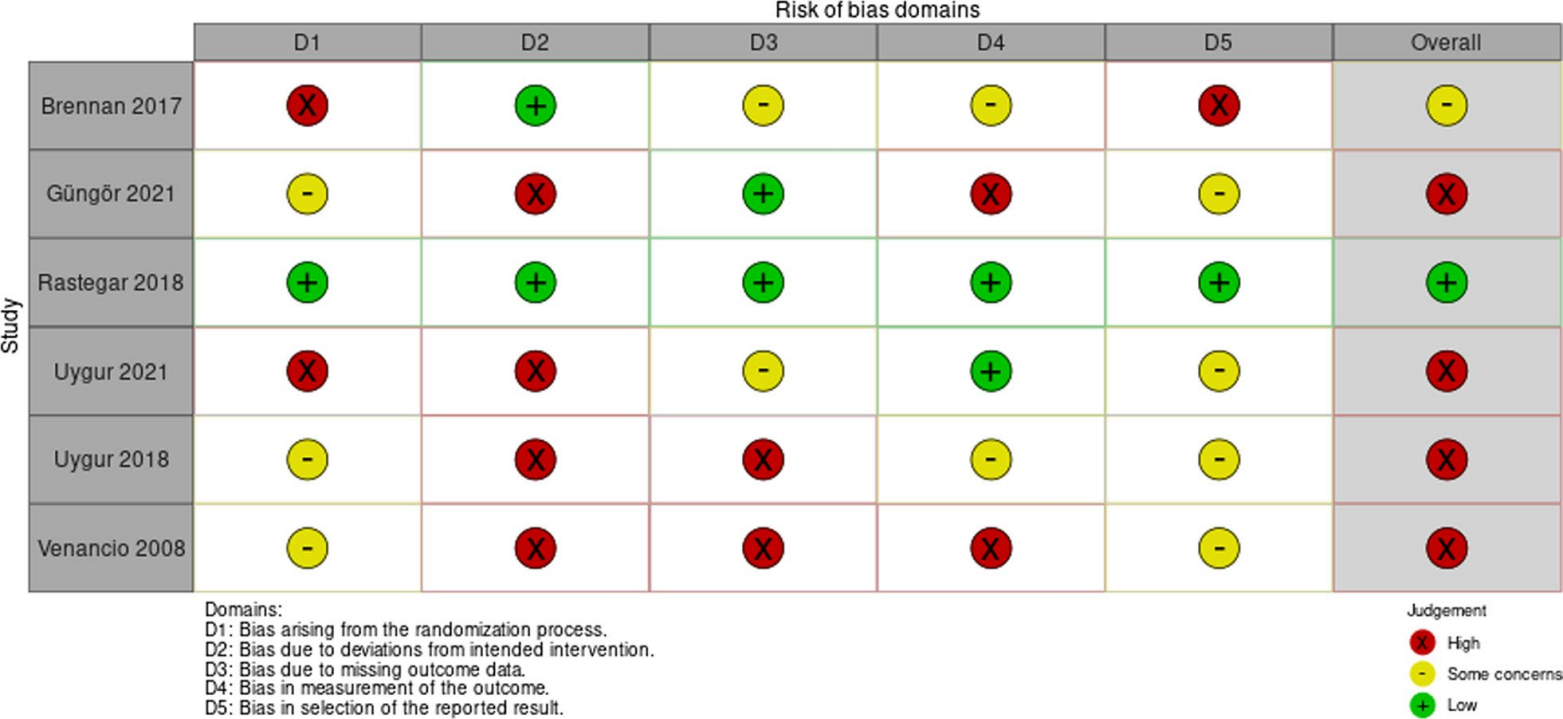
Risk of bias summary

The risk of bias is shown in Fig. 2. A good inter-observer agreement was observed (intraclass correlation coefficient: 0.86) and disagreements were resolved by discussion between first and second investigators. The randomization process was categorized as being at a low risk of bias for one trial [27], at a high risk of bias for two trials [26, 30] and with some concerns for three trials [28, 29, 31]. The description of the allocation concealment was unclear for most trials. Four studies exhibited high risk of bias due to deviations from intended intervention [28–31] and two exhibited high risk of bias for missing outcome data [28, 29]. Two studies presented high risk of bias for measurement of the outcome [29, 31] and four studies presented some concerns for selection of the reported result [28–31]. Brennan et al. [26] presented an outcome that was not pre-specified in the study registry, which indicates a selective reporting. Two trials presented some bias related to missing sample size calculation along with insufficient study reporting [29] and allowing co-intervention for only one treatment group [28]. Overall, four studies were judged as at a high risk of bias [28–31], one study was considered to present some concerns [26] and another was judged as at low risk of bias [27]. One study was judged as at low risk of bias for all five risk of bias domains [27].

The quality of evidence for each comparison is detailed in Table 2. All studies reported results for the short-term analysis. At 1 week, Brennan et al. [26] (DN 3.6 ± 2.1 vs CSI 2.6 ± 2.7) and Venancio et al. [29] (DN 0.34 ± 0.08 vs CSI 0.51 ± 0.20) have found no difference between DN and CSI for greater trochanteric and myofascial pain/headache, respectively. At 3 weeks, whereas Uygur et al. [28] (3wk: DN 27.7 ± 9.82 vs CSI 33.6 ± 10.6) and Brennan et al. [26] (DN 4.0 ± 2.2 vs CSI 2.7 ± 2.9) found no significant between-group difference for heel (plantar fasciitis) and greater trochanteric pain, respectively, Rastegar et al. [27] (DN 3.47 ± 1.32 vs CSI 0.32 ± 0.71) found more reduction in heel pain (plantar fasciitis) for CSI group. Even although Güngör et al. [31] found no difference between dry needling and corticosteroid injection (DN 2.3 ± 0.6 vs CSI 2.3 ± 0.6) for people with lateral epicondylitis at 3 weeks, Uygur et al. [30] found a significant difference in favor of dry needling at 20^th^ day follow-up (DN 15.7 ± 7.7 vs CSI 36.0 ± 14.7). At 4 weeks, Venancio et al. [29] (DN 0.42 ± 0.08 vs CSI 0.43 ± 0.11) found no differences between DN and CSI for myofascial pain/headache. At 6 weeks, Brennan et al. [26] (DN 2.8 ± 2.4 vs CSI 3.9 ± 3.7) found no between-groups difference for greater trochanteric pain, but Rastegar et al. [27] (DN 2.66 ± 1.33 vs CSI 0.21 ± 0.67) observed a significant heel pain (plantar fasciitis) reduction in favor of CSI in comparison to DN. The quality of the evidence was rated as very low-quality (GRADE).

**Table 2:** Quality of evidence according to GRADE approach

Outcome	Participants (studies)	Risk of bias	Inconsistency	Indirectness	Imprecision	Publication bias	Overall Quality of evidence
Pain (short-term)	384 (6 trials)	Serious	Serious	Serious	Serious	NA	Very low
Pain (medium-term)	144 (3 trials)	Serious	Serious	Not serious	Serious	NA	Very low
Pain (long-term)	263 (3 trials)	Serious	Serious	Serious	Serious	NA	Very low
Disability (short-term)	187 (3 trials)	Serious	Serious	Serious	Serious	NA	Very Low
Disability (medium-term)	48 (1 trial)	Serious	Serious	Serious	Serious	NA	Very low
Disability (long-term)	96 (1 trial)	Serious	Serious	Serious	Serious	NA	Very low

*NA* not applicable

Three studies assessed medium-term effects [27, 29, 31]. At 12 weeks, Venancio et al. [29] (DN 0.36 ± 0.17 vs CSI DN 0.33 ± 0.12) found no differences between DN and CSI for myofascial/headache pain and Rastegar et al. [27] (DN 1.59 ± 1.24 vs CSI 0.56 ± 1.33 p = 0.44) found that CSI is more effective than DN for reducing heel pain (plantar fasciitis). Güngör et al. [31] found a significant difference in favor of corticosteroid injection for people with lateral epicondylitis at 12 weeks follow-up (DN 1.16 ± 0.5 vs CSI 0.7 ± 0.6). The quality of the evidence was rated as very low-quality (GRADE).

Three studies reported results for long-term analysis [27, 28, 30]. Two studies were related to heel pain (plantar fasciitis). Uygur et al. [28] have found that DN was superior to CSI for reducing pain at 6 months follow-up (DN 29.7 ± 10.3 vs CSI 50.5 ± 12.3). Although Rastegar et al. [27] found no significant difference between DN and CSI in reducing pain at 6 months follow-up (DN 1.28 ± 1.46 vs CSI 1.79 ± 1.55 p = 0.65), there was a significant difference in favor of DN at 1-year follow-up (DN 0.69 ± 0.93 vs CSI 2.09 ± 1.58). At 6 months follow-up, Uygur et al. [30] found a significant difference in favor of dry needling (DN 9.7 ± 7.6 vs CSI 19.3 ± 19.4) for people with lateral epicondylitis. There is very low-quality evidence (GRADE) that DN was superior to CSI in pain reduction at long-term.

For disability, three distinct studies reported data at short term and the quality of evidence was considered to be very low (GRADE) [26, 28, 31]. Brennan et al. [26] evaluated disability in individuals with greater trochanteric pain syndrome at 1 week (DN 5.2 ± 2.2 vs CSI 6.5 ± 2.8), 3 weeks (DN 5.7 ± 2.0 vs CSI 6.5 ± 2.8) and 6 weeks (DN 7.3 ± 2.3 vs CSI 6.1 ± 3.0). No significant between-groups difference was observed. Uygur et al. [28] have also found no significant differences between DN and CSI (DN − 28.3 ± 8.9 vs CSI − 28.4 ± 11.6) for disability in plantar fasciitis at 3 weeks. Güngör et al. [31] assessed disability for people with lateral epicondylitis at 3 weeks and have found no between-group difference between dry needling and corticosteroid injection (DN 31.6 ± 6.8 vs CSI 32.0 ± 5.0).

One study presented mid-term results for disability [31]. Corticosteroid injection was superior to dry needling for people with lateral epicondylitis at 3 months follow-up (DN 30.0 ± 6.7 vs CSI 26.6 ± 3.2) [31]. For the long-term results, Uygur et al. [28] found a greater reduction of disability in DN group compared to CSI group (DN − 28.8 ± 8.8 vs CSI − 43.1 ± 11.1) for individuals with plantar fasciitis at 6 months follow-up. The quality of evidence was rated as very low-quality (GRADE).

## Discussion

Whereas dry needling and corticosteroid injections appear to present the same effect on pain and disability for people with myofascial pain and greater trochanteric pain syndrome, these interventions are likely to present distinct effects for heel pain (plantar fasciitis) and lateral epicondylitis. At short- and medium-term, corticosteroid injection seems to be superior to dry needling for reducing pain and disability in musculoskeletal conditions. At long-term, dry needling seems to be more effective than corticosteroid injection. However, the quality of evidence behind these findings is very low. In order to improve clinical decision making between dry needling and corticosteroid injection for musculoskeletal conditions, some important considerations regarding our results need to be taken into account.

Most included studies were at high risk of bias, which may affect the strength of the results. Although most studies presented small sample size, five studies calculated sample size for an α level of 0.05 and 80% power [26–28, 30, 31]. One study did not describe sample size calculation and presented a small sample size, which indicates it is very likely to be underpowered [29]. Evidence from this study was considered to be of poor quality because underpowered trials tend to over-estimate effect sizes [32]. Blinding of participants and personnel and allocation concealment were the main sources of bias. A previous review investigating the effectiveness of dry needling for musculoskeletal conditions has found blinding of therapists and subjects, intention-to-treat analysis and concealed allocation as the main sources of bias [11]. Another meta-analysis observed that the most common sources of bias among the studies verifying the effectiveness of corticosteroid injection for plantar heel pain were blinding of participants/personnel and outcome assessors [33]. While blinding of participants and therapists is typically not feasible for studies included in our systematic review due to the nature of interventions, concealed allocation should be adequately implemented and described in order to avoid selection bias. Although some studies did not observe significant influence of allocation concealment on treatment estimates of several interventions [34–36], there is no recommendation to stop using allocation concealment as a bias indicator because other previous works showed that this quality indicator may over- or underestimate the treatment effects [37, 38]. In addition, inadequate allocation concealment and lack of blinding contribute to an increased between-trial heterogeneity in meta-analyses studies [39, 40]. The high level of heterogeneity across studies in our systematic review prevented us from conducting a meta-analysis.

Despite the low methodological quality, our results indicate that there is no significant difference between CSI or DN for reducing pain at short-term follow-up (≤ 6 weeks) in individuals with greater trochanteric and myofascial pain/headache [26, 29]. This finding is intriguing because, if both interventions provide the same effect, clinical decision-making could consider other factors beyond interventions effect on pain such as adverse effects, cost or patient preference. However, the results may be interpreted with caution because the effectiveness of these interventions against placebo is still controversial. At the short-term, one study found effects in favor of corticosteroid injection at 3 weeks and 6 weeks for reducing pain in individuals with plantar fasciitis when compared to dry needling [27]. This study showed outcomes that achieved the minimal clinically important difference (MCID), which is two points out of ten on an 11-points scale for chronic pain conditions [41], and was considered to be at a lower risk of bias than the others were. At the medium-term (7–23 weeks) follow-up, this same study found effects in favor of corticosteroid injection, but these effects were not clinically significant. Whereas one study has found that DN is superior to CSI for lateral epicondylitis at short-term [30], another study has observed no difference between DN and CSI at short-term and a significant effect in favor of CSI at medium-term follow-up [31]. Both studies were at high risk of bias. Therefore, we are not sure if the observed overall short- and medium-term effects would remain the same if studies with higher methodological quality were added to the analysis.

At long-term, dry needling seems to be more effective than corticosteroid injection for reducing heel pain and lateral elbow pain. The between-groups difference found in favor of dry needling in the study assessing people with lateral epicondylitis achieved a minimal clinical significance, which is seven points out 100 in the PRTEE tool [30, 42]. Two studies included in this review investigated the effects of DN and CSI for plantar fasciitis [27, 28]. Although Rastegar et al. [27] found a significant difference in favor of DN at 1-year follow-up, the achieved reduction in pain levels was not clinically important (MCID < 2 points). Uygur et al. [28] found a significant pain reduction in DN group in comparison to CSI group at 6 months follow-up. This difference achieved the minimal important difference (MID) for people with plantar fasciitis, which is 6.5 points change in the Foot function index (FFI) [43]. While a previous meta-analysis has found that corticosteroid injections are not different from placebo for pain outcomes [34], a RCT has showed that dry needling is superior to sham dry needling in individuals with plantar heel pain [44] at a 12 weeks follow-up. As placebo effect may reduce over the time, especially because most corticosteroid injections are delivered by one single application and dry needling intervention generally is performed in multiple treatment sessions, it is reasonable to hypothesize that dry needling would show better results than corticosteroid injection at longer follow-up periods. In fact, previous trials have found that corticosteroid injection tends to present either similar or greater effects at short-term than long-term for some comparators and no differences at the long-term [3, 34]. To ensure that the superiority effect from dry needling at long-term is derived from the intervention itself, large RCTs with adequate power and methodological quality may be performed.

For disability, there was no difference between dry needling and corticosteroid injection for greater trochanteric pain syndrome, plantar fasciitis or lateral epicondylitis at short-term. It is important to note that one study investigating people with plantar fasciitis [28] presented effects on disability that are similar to that observed for pain levels, where dry needling appeared to be more effective than corticosteroid injection for reducing disability at longer follow-up periods. The similarity between pain and disability results may be due to the clinical relationship among these variables. It has been observed that reduction in pain levels lead to reduction in disability scores for individuals with musculoskeletal conditions [45]. As cognitive-emotional factors may mediate this response, when musculoskeletal pain intensity is reduced along with improvements in pain acceptance or catastrophizing, the individuals would feel more confident and fearless to perform activities of daily living, thus reducing disability. Although disability is an important outcome, only half of the included studies reported disability as an outcome. Musculoskeletal conditions are the leading cause of disability worldwide. Therefore, it is highly recommended that future studies include disability as an outcome.

Both corticosteroid injection and dry needling are suggested to reduce pain and disability from distinct mechanisms. While dry needling effects are derived from the needling stimulation over the tissue, corticosteroid injection effects are derived from the action of the drug that is being injected [46]. Dry needling elicits a local twitch response that emits neural signals capable of breaking the vicious cycle of pain-spasm-pain [8, 46]. As there is no consensus regarding needling duration, frequency, location and intensity [8], previous studies have adopted different needling procedures to assess the effects of dry needling for musculoskeletal pain and functional outcomes [11]. In our review, dry needling intervention was applied under different protocols, which would contribute to high heterogeneity across studies. For this reason, meta-analysis was not performed and we were not able to examine to which extent the differences in dry needling application across the studies are likely to influence the outcomes. It is reasonable to suggest that dry needling results could be favored by the increased contact between patient-therapist. Whereas CSI was performed in one single session, DN intervention was delivered in up to 7 sessions. As the patient-therapist interaction may be a source of implicit bias, it may be considered when interpreting the results [47].

Corticosteroid injection action primarily depends on the mechanism of corticosteroid drugs. Usually, these drugs act by suppressing release or activation of proinflammatory cytokines such as interleukins, chemokines and prostaglandins. Three studies in our review have used a particulate steroid (methylprednisolone acetate), while one study used a non-particulate steroid (dexamethasone). Although non-particulate steroids are short-acting and more soluble than particulate steroids, we believe that the type of injected steroid would not influence our findings because the only study that has used non-particulate steroid just assessed short- and medium-term effects [48]. Thus, the effects of the short-acting steroid would be still present in these time points. Ongoing studies may better clarify if there is difference between particulate and non-particulate steroids for common musculoskeletal conditions.

This study has some limitations. Only six studies were included in the review, which limits the comparisons, reduces the strength of the results, and weakens the generalization of the findings. In addition, the treatment estimates presented substantial heterogeneity, which prevented us from conducting a meta-analysis. Furthermore, the methodological quality of the studies was very low quality. Therefore, we emphasize the need for large RCTs with adequate sample size and statistical power as well as adequate study reporting with proper description of allocation concealment, blinding, and intervention procedures.

## Conclusions

In conclusion, there is very low quality of evidence that there is no significant difference between CSI or DN for pain or disability at short-, medium- or long-term follow-up in people with myofascial pain and greater trochanteric pain syndrome. Very low-certainty evidence suggests that corticosteroid injection is superior to dry needling at shorter follow-up and dry needling is more effective than corticosteroid injection at long-term follow-up for reducing pain and/or disability in people with plantar fasciitis and lateral epicondylitis. Large RCTs with higher methodological quality are needed in order to draw more incisive conclusions.

Clinicians and researchers may be aware that although both interventions presented effects for pain at short-, medium-, and long-term follow-up in the assessed musculoskeletal conditions, these findings are supported by insufficient evidence. Therefore, we suggest that corticosteroid injections and dry needling should be used with caution in the clinical settings and when making a clinical decision about which intervention to use, the professionals should also consider other factors such as adherence, costs, patient preference and adverse events. The finding that CSI seems to be more effective than DN at shorter follow-ups and DN appears to be superior to CSI at long-term follow-up for the management of heel and lateral elbow pain and that DN and CSI seems to present similar effects for the management of myofascial and greater trochanteric pain may help clinicians to make informed clinical choices when choosing to use these interventions as adjunct therapies.

## Supplementary Information

**Additional file 1**. Search strategy.

## Data Availability

All data generated or analyzed during this study are included in this published article [and its Additional file 1].
