# Supplementary material for: Corticosteroid injection or dry needling for musculoskeletal pain and disability? A systematic review and GRADE evidence synthesis
Source: Chiropr Man Therap. 2021 Dec 2;29:49. doi: 10.1186/s12998-021-00408-y (PMC8638538; doi:10.1186/s12998-021-00408-y)
Supplement: Supplementary file 1 — Additional file 1. Search strategy. [file 12998_2021_408_MOESM1_ESM.docx]

**APPENDIX – Search Strategy**

**MEDLINE (Pubmed) 1916 to Present (*31 October 2021*) n=668**

1. “dry needling” [MeSH Terms]

2. “dry needling” [Title/Abstract]

3. “intramuscular stimulation” [All fields]

4. Needling [Title/Abstract]

5. Needles [Title/Abstract]

6. “intramuscle stimulation” [Title/Abstract]

7. “dry needle” [Title/Abstract]

8. “dry needl*” [Title/Abstract]

9. #1 OR #2 OR #3 OR #4 OR #5 OR #6 OR #7 OR #8

10. corticosteroid* [Title/Abstract]

11. injection* [Title/Abstract]

12. steroids [MeSH Major Topic]

13. corticosterone [MeSH Terms]

14. “adrenal cortex hormones” [MeSH Terms]

15. #10 OR #11 OR #12 OR #13 OR #14

16. “randomized controlled trial” [Publication Type]

17. “controlled clinical trial” [Publication Type]

18. “clinical trial” [Publication Type]

19. “clinical study” [Publication Type]

20. random* [Title/Abstract]

21. “trial” [Title/Abstract]

22. #16 OR #17 OR #18 OR #19 OR #20 OR #21

23. #9 AND #15 AND #22

**WEB OF SCIENCE (Clarivate analytics) 1945 to Present (*31 October 2021*) n=163**

1. TS=("dry needling")

2. TI=("intramuscular stimulation")

3. TI=(needles)

4. TI=(needling)

5. #1 OR #2 OR #3 OR #4

6. TS=(corticosteroid*)

7. TI=(corticosteroid*)

8. AB=(corticosteroid*)

9. KP=(steroids)

10. #6 OR #7 OR #8 OR #9

11. #5 AND #10

**SCOPUS (Elsevier) 1960 to Present (*31 October 2021*) n=269**

1. TITLE-ABS-KEY("dry needling")

2. TITLE-ABS-KEY(“intramuscular stimulation”)

3. TITLE-ABS-KEY(needles)

4. TITLE-ABS-KEY(needling)

5. #1 OR #2 OR #3 OR #4

6. TITLE-ABS-KEY(corticosteroid*)

7. TITLE-ABS-KEY(corticosterone)

8. TITLE-ABS-KEY(steroids)

9. #6 OR #7 OR #8

10. TITLE-ABS-KEY(“randomized controlled trial”)

11. TITLE-ABS-KEY(“controlled clinical study”)

12. TITLE-ABS-KEY(“randomized clinical trial”)

13. SRCTYPE (random*)

14. #10 OR #11 OR #12 OR #13

15. #5 AND #9 AND #14

**SPORTDISCUS (EBSCO) 1800 to Present (*31 October 2021*) n=150**

1. TI “dry needling”

2. AB “dry needling”

3. TI “intramuscular stimulation”

4. AB “intramuscular stimulation”

5. TI “needles”

6. AB “needles”

7. TI “needling”

8. AB “needling”

9. #1 OR #2 OR #3 OR #4 OR #5 OR #6 OR #7 OR #8

10. TI “corticosteroid*”

11. AB “corticosteroid*”

12. TI “corticosterone”

13. AB “corticosterone”

14. TI “steroids”

15. AB “steroids

16. TI “injection”

17. AB “injection”

18. #10 OR #11 OR #12 OR #13 OR #14 OR #15 OR #16 OR #17

19. #9 AND #18

**CINAHL (EBSCO) 1937 to Present (*31 October 2021*) n=49**

1. MM “dry needling”

2. TI “dry needling”

3. AB “dry needling”

4. TI “intramuscular stimulation”

5. AB “intramuscular stimulation”

6. TI “needles”

7. AB “needles”

8. TI “needling”

9. AB “needling”

10. TI “dry needl*”

11. AB “dry needl*”

12. #1 OR #2 OR #3 OR #4 OR #5 OR #6 OR #7 OR #8 OR #9 OR #10 OR #11

13. TI “corticosteroid*”

14. AB “corticosteroid*”

15. TI “corticosterone”

16. AB “corticosterone”

17. TI “steroids”

18. AB “steroids”

19. TI “injection”

20. AB “injection”

21. #13 OR #14 OR #15 OR #16 OR #17 OR #18 OR #19 OR #20

22. #12 AND #21

**MEDLINE** 668 articles

**SCOPUS** 269 articles

**WEB OF SCIENCE** 163 articles

**CINAHL** 49 articles

**SPORTDiscus** 150 articles

**Total** 1,299 articles

***GREY LITERATURE – OpenGrey (n=5) and Oaister (n=1,060) – (31 October 2021)***

1. “dry needling”

2. “dry needl*”

3. “intramuscular stimulation”

4. needles

5. needling

6. #1 OR #2 OR #3 OR #4 OR #5

7. corticosteroid*

8. steroids

9. injection

10. #7 OR #8 OR #9

11. #6 AND #10
